# Mobile phone addiction in African societies

**DOI:** 10.3389/fpsyg.2026.1773697

**Published:** 2026-04-20

**Authors:** Thabani Khumalo

**Affiliations:** Discipline of Psychology, School of Social Sciences, University of KwaZulu-Natal, Pietermaritzburg Campus, Durban, South Africa

**Keywords:** Africa, cross-cultural assessment, decolonizing psychology, problematic smartphone use, smartphone addiction, South Africa, *Ubuntu*

## Abstract

Smartphone adoption has risen rapidly across Africa, yet psychological models of problematic smartphone use (PSU) derive mainly from samples drawn from Western, Educated, Industrialized, Rich, and Democratic (WEIRD) populations and from individualistic assumptions. In collectivist settings, *Ubuntu* philosophy; “I am because we are,” frames communication as a relational duty rather than a purely individual choice. This Perspective contends that behaviors often labeled “excessive” under Western criteria (e.g., high-frequency family WhatsApp communication, rapid group responsiveness) may represent normative expressions of relationality in African contexts, risking the pathologization of *Ubuntu*-aligned communication norms. We advance three propositions: (1) extended family communication reflects *Ubuntu* relationality rather than addiction; (2) the fear of missing out (FOMO) operates differently where communal obligations are primary; and (3) widely used instruments (e.g., SAS/SAS-SV) may misclassify *Ubuntu*-aligned behaviors as dysfunction due to embedded cultural bias. We also identify contextual determinants (load-shedding, prepaid data constraints, communal device use) that shape patterns of smartphone use. We present a research agenda for culturally appropriate measurement, contextual investigation, and *Ubuntu*-informed interventions. This Perspective contributes to the decolonization of psychological assessment, advancing cultural validity in psychological science with implications for theory, clinical practice, public health, and technology design.

## 1 Introduction

Smartphone use is now embedded in daily life worldwide; in South Africa, household smartphone penetration exceeds 90% (Elad and Jambhale, 2025; Mostert, 2025). As access expands, psychology has increasingly focused on problematic smartphone use (PSU); persistent difficulty regulating use with negative consequences (Billieux, 2012; De-Sola Gutiérrez et al., 2016). Yet PSU theory and assessment are overwhelmingly derived from Western, Educated, Industrialized, Rich, and Democratic (WEIRD) populations (more than 95% of studies), while Africa contributes just less than 1% of samples despite comprising 17% of humanity (Barrett, 2020; Nielsen et al., 2017; Rosales, 2024).

We contend this imbalance threatens construct validity: instruments calibrated to individualistic values may misread collectivist practices. Emerging cross-cultural evidence shows that high interaction frequency and fast responsiveness can be relationally appropriate rather than compulsive (Olson et al., 2023; Soraci et al., 2024). Accordingly, we reframe PSU through *Ubuntu*, which emphasizes interconnectedness, communal identity, and responsibility (Ajitoni, 2024; Mugumbate et al., 2023; Nzimakwe, 2014; Rotzinger et al., 2025). Our aims are to (a) argue that certain smartphone behaviors are normative relational practices; (b) specify measurement risks of cultural misclassification; and (c) outline a pragmatic agenda for culturally valid assessment and intervention.

## 2 Problem statement

The systematic application of Western-derived problematic smartphone use (PSU) assessment instruments in African collectivist contexts creates a fundamental measurement validity crisis with documented empirical consequences. Current diagnostic frameworks, developed primarily from WEIRD samples representing less than 12% of the global population (Henrich et al., 2010), are being applied to the remaining 88% of humanity without adequate cultural validation (Barrett, 2020; Nielsen et al., 2017). This practice threatens the integrity of prevalence estimates, clinical decision-making, and intervention research across the African continent.

Recent epidemiological data reveal the magnitude of this problem. Global PSU prevalence estimates range from 10 to 28% depending on the instrument used and the population sampled (Olson et al., 2023), but these figures derive almost exclusively from samples in East Asia, Europe, and North America. African studies constitute less than 1% of the PSU research literature despite Africa comprising 17% of the world’s population (Rosales, 2024). When Western instruments are deployed in African settings, they produce prevalence estimates exceeding 30% among university students (Mostert, 2025), rates substantially higher than those observed in WEIRD populations using identical cutoff scores. Critically, this pattern extends beyond Africa: a comprehensive meta-analysis of 63 independent samples with 34,798 respondents from 32 nations revealed that the pooled prevalence estimate in collectivist nations (31, 95% CI: 26–36%) was twofold higher than in individualist nations (14, 95% CI: 9–19%) (Leung et al., 2020). This systematic disparity across multiple world regions strongly suggests that measurement bias, not genuine dysfunction, drives observed differences.

The specific mechanism of misclassification centers on the cultural interpretation of what we term connectivity behaviors: socially embedded, goal-directed digital practices through which individuals initiate, sustain, and fulfill relational obligations within their social networks (Alshare et al., 2024; Olson et al., 2023). Instruments such as the Smartphone Addiction Scale (SAS) and its short version (SAS-SV) operationalize excessive use through items that assess frequency of checking, inability to reduce use, and distress when separated from the device (Kwon et al., 2013). However, these items fail to distinguish between individual compulsion and relational obligation. In *Ubuntu*-oriented cultures, where personhood is defined relationally through the principle “I am because we are” (Mugumbate and Chereni, 2020), high-frequency checking of family WhatsApp groups, rapid responsiveness to elder requests, and distress when unable to fulfill communication duties are normative expressions of moral responsibility rather than symptoms of addiction. Recent empirical work reinforces this interpretation: individuals from collectivistic societies are more involved in using social media to share feelings and ideas, producing higher smartphone addiction scores, but this intensive use reflects cultural communication norms rather than pathology (Alshare et al., 2024). A 2025 study in Turkey found that FOMO operated through pathways related to maintaining social cooperation, with smartphone use serving to sustain culturally valued face-to-face interactions rather than indicating individual dysfunction (Tufan et al., 2025). Cross-cultural research involving 5,900 students from seven countries including South Africa confirmed that endorsement of collectivism versus individualism strongly predicts emotion regulation and social behaviors, but PSU instruments fail to account for these cultural differences (Klein et al., 2024).

The consequences of this misclassification are measurable and significant. First, inflated prevalence estimates misrepresent the scope of genuine dysfunction, leading to misallocation of limited mental health resources in settings where clinician-to-population ratios are already critically low. Second, individuals exhibiting culturally normative relational behaviors risk being labeled as pathological, potentially triggering stigma, unnecessary clinical referrals, and interventions that undermine rather than support adaptive social functioning. Third, research findings based on these flawed measurements produce distorted knowledge about the nature, causes, and correlates of PSU in non-WEIRD populations, perpetuating an evidence base that is epistemically unjust and practically inadequate for guiding intervention development. Fourth, the absence of culturally valid measurement tools precludes meaningful cross-cultural comparison and prevents African scholars from contributing authoritative knowledge about digital behavior in their own contexts.

## 3 Purpose of the study

The purpose of this conceptual analysis is to critically interrogate the cultural validity of Western-derived problematic smartphone use (PSU) constructs when applied to African collectivist contexts, and to reconceptualize smartphone connectivity through *Ubuntu* philosophy as an interpretive framework that can distinguish relational obligation from individual compulsion. This work responds to the documented absence of culturally grounded measurement in PSU research, where 99% of studies derive from WEIRD populations despite Africa’s substantial and rapidly growing smartphone user base (Barrett, 2020; Nielsen et al., 2017; Rosales, 2024).

Specifically, this study seeks to accomplish three interconnected objectives. First, it aims to interrogate the applicability of Western-derived PSU constructs, particularly those operationalized in widely used instruments such as the Smartphone Addiction Scale (SAS/SAS-SV), in African cultural contexts where *Ubuntu* philosophy shapes the meaning and enactment of digital communication. Second, this study aims to demonstrate how *Ubuntu* philosophy reframes interpretations of high-intensity digital connectivity by repositioning the unit of analysis from the individual to the relational web in which personhood is constituted. Third, this study aims to propose directions for culturally grounded assessment and intervention by outlining a research agenda that centers African epistemologies, participatory methodologies, and community-based validation processes.

It is crucial to clarify what this study does not attempt to accomplish. This is not an empirical investigation that measures PSU prevalence, tests hypotheses about risk factors, or evaluates intervention efficacy. Rather, it is a conceptual and critical analysis that operates at the level of construct validity, measurement theory, and epistemic justice. The intended contribution is threefold: theoretically, it advances understanding of how cultural meaning systems shape digital behavior; methodologically, it identifies specific sources of measurement bias in cross-cultural PSU research; and practically, it offers guidance for clinicians, researchers, and policymakers working in African contexts.

## 4 Nature of inquiry

This study employs a conceptual and critical approach to inquiry, situated at the intersection of cultural psychology, indigenous philosophy, and critique of measurement science. The nature of this investigation is fundamentally interpretive rather than empirical, focusing on the analysis and reconceptualization of existing constructs rather than the collection and statistical analysis of new data. This methodological positioning is deliberate and appropriate to the study aims: to interrogate the cultural validity of Western-derived frameworks, to demonstrate how *Ubuntu* philosophy offers an alternative interpretive lens, and to generate theoretical foundations for future empirical research.

The epistemological stance of this inquiry aligns with social constructionist and cultural-ecological traditions in psychology, which recognize that psychological phenomena are not culturally neutral objects awaiting discovery but are constituted through meaning-making processes embedded in distinct social, historical, and material contexts (Bruner, 1990; Cole, 1996). From this perspective, problematic smartphone use is not a universal category with fixed boundaries that can be measured identically across cultures. The inquiry therefore adopts a stance of epistemic humility regarding Western frameworks while centering *Ubuntu* philosophy as an equally legitimate, and arguably more ecologically valid, foundation for understanding smartphone behavior in African collectivist contexts.

Methodologically, this study draws on several complementary approaches. First, it employs conceptual analysis to examine the implicit assumptions embedded in Western PSU frameworks. Second, it engages in philosophical exegesis of *Ubuntu* literature to articulate the relational ontology, moral framework, and social psychology implied by the principle “I am because we are.” Third, it undertakes theoretical integration to show how *Ubuntu* philosophy can function as an analytic lens that reinterprets behaviors currently labeled as pathological.

## 5 Significance of the study

This study addresses a problem of substantial theoretical, clinical, and social significance that affects millions of smartphone users across African contexts and challenges the epistemological foundations of cross-cultural psychological science. The significance can be understood through three interconnected dimensions: its contribution to scientific knowledge, its implications for professional practice, and its potential impact on the wellbeing of individuals and communities currently subject to culturally inappropriate assessment and intervention.

Theoretically, this study fills a critical gap in the PSU literature by providing the first systematic conceptual framework for understanding smartphone use in African collectivist contexts through indigenous philosophy. Despite rapid smartphone adoption across Africa, with penetration exceeding 90% in many urban centers (Elad and Jambhale, 2025), the continent contributes less than 1% of PSU research samples (Rosales, 2024). This study addresses this gap not by simply adding African samples to Western frameworks, but by demonstrating that such frameworks are conceptually inadequate for capturing the relational dynamics that organize digital behavior in *Ubuntu*-oriented societies.

Practically, the study has direct implications for clinical psychology, counseling, and mental health service delivery across African contexts. Mental health professionals working in these settings face a persistent dilemma: the assessment tools available to them are designed for Western populations and may not validly identify dysfunction in collectivist contexts. This study provides clinicians with a framework for contextualizing intensity within relational obligations, distinguishing adaptive connectivity from genuine pathology, and recognizing when interventions aimed at reducing smartphone use would undermine rather than support clients’ social functioning.

For affected individuals and communities, the study’s significance lies in its potential to reduce stigma, prevent misdiagnosis, and ensure that mental health interventions are culturally appropriate. When university students, employees, or community members scoring high on Western PSU instruments are labeled as addicted despite functioning well in their social roles, this creates unnecessary distress and undermines confidence in mental health services. The study provides an alternative interpretive framework that honors *Ubuntu* values and treats relationality as a strength rather than a symptom.

## 6 Theoretical framework

This study integrates *Ubuntu* philosophy with established psychological theories to create a comprehensive framework for understanding smartphone use in African collectivist contexts. While *Ubuntu* serves as the primary philosophical foundation, its integration with Social Ecological Theory, Cultural Models Theory, and Role Theory provides conceptual bridges to mainstream psychological science while preserving the distinctiveness of African relational ontology.

### 6.1 Social Ecological Theory

Social Ecological Theory (Bronfenbrenner, 1979; Sallis et al., 2008) posits that human behavior is shaped by multiple, nested levels of influence: individual, interpersonal, community, institutional, and structural systems. This theory applies powerfully to this manuscript because it supports the argument that smartphone use behaviors cannot be interpreted solely at the individual level, the focus of most Western PSU frameworks. The paper’s discussion of load-shedding, data precarity, communal device sharing, and economic survival functions maps directly onto ecological levels beyond the individual.

### 6.2 Cultural Models Theory

Cultural Models Theory, rooted in cognitive anthropology and cultural psychology (Quinn and Holland, 1987; Strauss, 1992), argues that people rely on culturally shared schemas to interpret behavior, norms, and meaning. *Ubuntu* functions as a cultural model that defines responsiveness, availability, and communication frequency as moral and relational obligations rather than discretionary behaviors. This theory helps explain why Western PSU instruments systematically misclassify *Ubuntu*-aligned behaviors: they operationalize dysfunction using a cultural model of autonomy that does not align with collectivist schemas.

### 6.3 Role Theory

Role Theory (Biddle, 1986; Merton, 1957) examines how social roles, expectations, and norms guide behavior. In *Ubuntu*-oriented societies, individuals occupy multiple roles within extended family and community networks, each carrying communication obligations. From a Role Theory perspective, frequent smartphone checking reflects role maintenance: monitoring family WhatsApp groups ensures one remains informed of obligations. The concept of FOMO transforms from individual status anxiety to fear of failing communal obligations, what might be termed fear of failing others (FFO).

## 7 Integration with *Ubuntu* philosophy

These theories do not supplant *Ubuntu* but rather provide conceptual bridges that help translate *Ubuntu* philosophy into constructs recognizable within mainstream psychological science. Where Western individualism treats the autonomous self as the basic unit of analysis, *Ubuntu* treats the relational web as primary. By integrating *Ubuntu* with Social Ecological, Cultural Models, and Role theories, the framework honors both the distinctiveness of African relational ontology and the accumulated knowledge of psychological science.

### 7.1 *Ubuntu* philosophy as a psychological lens

*Ubuntu*, “a person is a person through other people,” centers communal obligations and relational identity (Mugumbate and Chereni, 2019; Ajitoni, 2024). Empirical work indicates these values persist among emerging adults in Namibia and Kenya (Rotzinger et al., 2025). Psychologically, this means connectivity behaviors; frequent check-ins, rapid replies, group coordination, may represent relational duty rather than individual compulsion. The implication is that PSU criteria should weigh relational goals and obligation-fulfillment before inferring dysregulation.

## 8 Core propositions

This section advances three interrelated propositions that challenge the application of Western smartphone addiction frameworks in African contexts. Each proposition draws on *Ubuntu* philosophy to reinterpret behaviors that might otherwise be pathologized through individualistic lenses.

### 8.1 Extended family WhatsApp use reflects relationality, not addiction

Multi-generational, high-frequency WhatsApp communication in African contexts often expresses *Ubuntu* relational expectations rather than addictive behavior (Mugumbate and Chereni, 2019; Mugumbate et al., 2023). This distinction is crucial because intensity alone is insufficient for establishing pathology. Instead, clinicians and researchers must assess distress, role impairment, and negotiation failures within family systems (Billieux, 2012; De-Sola Gutiérrez et al., 2016). The interpretive shift here is significant: in African samples, messaging frequency may correlate with bonding and support at least as strongly as with dysfunction, suggesting patterns that likely diverge from WEIRD norms (Olson et al., 2023). This proposition calls into question the foundational assumption that high-frequency digital communication necessarily indicates problematic use.

It is important to clarify the theoretical distinctiveness of the *Ubuntu* framework relative to existing context-sensitive movements within Western PSU scholarship. Contemporary harm-based frameworks; including Billieux’s (2012) functional impairment model and ICD-11 criteria for compulsive behavior disorders, already attempt to correct the frequency fallacy by requiring that use produce significant negative consequences before pathological classification is warranted. These advances are genuinely important. However, they remain anchored in methodological individualism: the unit of assessment is the individual, and harm is measured as disruption to that individual’s functioning, relationships, and wellbeing. *Ubuntu* philosophy does not merely modulate this framework contextually; it challenges its foundational unit of analysis. Under *Ubuntu* ontology, the person is constituted through others: “I am because we are.” This means that the clinically relevant question is not whether high-frequency use disrupts individual functioning, but whether use disrupts or sustains the relational obligations through which personhood is enacted. A student who monitors family WhatsApp groups through the night to respond to an elder’s request may score highly on individual disruption indices (lost sleep, reduced study time) while simultaneously fulfilling core obligations through which their social identity and wellbeing are constituted. The *Ubuntu* framework therefore does not represent a contextual extension of individualist assessment; it reorients the telos of clinical judgment from individual autonomy regulation to relational obligation fulfillment, a shift with far-reaching consequences for how “harm” and “dysfunction” are defined and measured.

### 8.2 FOMO is communal in collectivist contexts

In its original Western theorization, fear of missing out (FOMO) is conceptualized as a pervasive apprehension that others might be having rewarding experiences from which the individual is absent, arising from unmet psychological needs for belonging, competence, and autonomy (Przybylski et al., 2013). Within this framework, FOMO is fundamentally an individual affective state: it signals self-determined deficit and is associated with social comparison, low life satisfaction, and compulsive social media monitoring. The construct assumes that the primary referent of distress is the individual self and its standing relative to peers. Building on this foundation, we argue that FOMO operates fundamentally differently in *Ubuntu*-oriented settings, where the primary referent is not the self but the relational web of obligations in which the self is constituted. Here, FOMO centers on fear of failing communal duties rather than individual status anxiety (Bakuri and Amoabeng, 2023); a distinction with direct implications for both assessment item content and intervention targets.

The figure below illustrates a two-panel contrast of interpretive lenses: individual dysregulation versus relational responsibility and social cohesion. The bridge between these panels indicates how identical behaviors; such as frequent family WhatsApp contact, can be misclassified depending on the interpretive lens applied (Table 1).

**Table 1:** Comparison of Western PSU framework and *Ubuntu* connectivity framework.

Dimension	Western PSU framework	*Ubuntu* connectivity framework
Core assumption	Behavior reflects individual dysregulation of self-control	Behavior expresses relational duty and communal identity
Meaning of high frequency messaging	Indicator of compulsive use or dependence	Normative fulfillment of relational expectations within extended family
Meaning of rapid responsiveness	Loss of control/compulsive checking	Respect, responsibility, and maintaining social harmony
FOMO (fear of missing out)	Anxiety about individual status, comparisons, and exclusion	Concern about failing obligations or disrupting group cohesion
Primary unit of analysis	The individual user	The relational network (family, community)
Interpretive risk	Over pathologizing normative social behaviors	Under-recognizing genuine distress due to contextual stressors
Assessment priority	Screen time, control, withdrawal, tolerance	Obligation alignment, relational strain, contextual constraints

From this perspective, we propose two *Ubuntu*-specific constructs for inclusion in a culturally grounded assessment instrument. Role-performance concerns refer to anxiety arising specifically from the perceived failure to fulfill named relational duties through digital communication; for example, anxiety about not monitoring family WhatsApp groups promptly, failing to respond to elders within culturally expected timeframes, or being absent from important communal digital events. These concerns would be operationalized through items such as: “I feel anxious when I am unable to respond to family messages in the way expected of me,” or “I worry that my unavailability on family WhatsApp groups reflects poorly on me as a family member.” Obligation misalignment refers to the experienced discrepancy between one’s actual responsiveness capacity and community-held norms around digital availability; a construct assessing not individual compulsion but structural mismatch between relational expectations and material constraints such as limited data access or network instability. Sample items might include: “I often cannot meet the communication expectations my family has of me due to circumstances beyond my control,” or “I feel I am letting my community down because I cannot always be as connected as expected.” These constructs are conceptually related to but distinct from individualist FOMO: they share the surface feature of distress at disconnection but differ in motivational basis (relational duty versus status anxiety), cognitive content (failing others versus missing out personally), and clinical implications (obligation repair and structural intervention versus self-regulation training). They are therefore proposed as core dimensions of the *Ubuntu* Smartphone Use Scale (USUS) rather than adaptations of existing FOMO scales, and their discriminant validity relative to established FOMO measures should be examined in future validation research (Przybylski et al., 2013; Terblanché-Greeff and Nel, 2023).

### 8.3 Western instruments misclassify *Ubuntu*-aligned behaviors

The third proposition addresses measurement directly. Widely used scales such as the Smartphone Addiction Scale (SAS) and its short version (SAS-SV) embed individualistic assumptions about self-regulation and may therefore pathologize collectivist norms (Kwon et al., 2013; Mostert, 2025). To illustrate this problem, Table 2 below presents a three-column mapping that connects representative SAS/SAS-SV item themes with reasons for their misclassification in African contexts and proposes *Ubuntu*-aligned constructs for the *Ubuntu* Smartphone Use Scale (USUS).

**Table 2:** Mapping measurement bias: SAS/SAS-SV vs. *Ubuntu*-informed constructs.

SAS/SAS-SV item theme	Why it misclassifies *Ubuntu*-aligned behavior	*Ubuntu*-informed USUS construct (proposed)
“*I miss planned work due to smartphone use*”	The phone is often used for coordinating collective responsibilities; interruptions may reflect communal demands, not addiction	Relational workload coordination; balancing collective obligations
“*I feel impatient and restless when I do not have my smartphone*”	Restlessness may reflect anxiety about missing obligations or family updates, not dependency	Obligation-related anticipatory stress
“*I constantly check my smartphone so as not to miss conversations*”	High responsiveness is a sign of respect and relational attentiveness	Communal responsiveness rhythm
“*I use my smartphone longer than I intend*”	Duration is shaped by shared devices, voice notes, and family group discussions; long use ≠ dysregulation	Shared-device interaction patterns and intergenerational communication
“*I have difficulty concentrating due to smartphone use*”	Multitasking may be a norm in high-responsibility communal roles	Relational multitasking strain
“*People complain about my smartphone use*”	“Complaints” differ culturally; some families expect high availability, others prefer in-person interaction	Obligation negotiation within family systems
*General Scale Logic (treats intensity as pathology)*	Intensity does not account for structural determinants (load-shedding, data scarcity, economic survival use)	Contextual adaptation (infrastructure, data, economic pressures)

It is essential to clarify what Table 2 does and does not imply. We do not argue that problematic smartphone use is absent in *Ubuntu*-oriented contexts, or that high-frequency use is categorically adaptive when it occurs within collectivist meaning systems. Compulsive, ego-dystonic, and harmful smartphone use can and does occur among *Ubuntu*-oriented individuals. Online gambling addiction among economically marginalized South African men (Rammutla, 2024), compulsive use patterns that individuals themselves report as distressing and contrary to their values, and smartphone behaviors that community members identify as disrupting family functioning are all examples of genuinely problematic use that a culturally sensitive framework must recognize and address. Rather than suggesting that PSU is absent, Table 2 argues that it manifests differently and requires distinct diagnostic criteria calibrated to relational rather than individual units of assessment. Within an *Ubuntu* framework, the following indicators signal genuinely problematic use warranting clinical concern: (a) use experienced by the individual as ego-dystonic—unwanted, distressing, and contrary to personally held values and relational commitments; (b) use that disrupts rather than sustains relational obligations, for example leading to neglect of care responsibilities, missed community events, or deterioration of important relationships; (c) use that produces community-recognized harm, particularly involving financial ruin, neglect of dependents, or social isolation from one’s relational network; and (d) use that the individual and their relational community jointly identify as requiring intervention, drawing on communal processes of accountability and care. These criteria preserve the relational unit of analysis while providing clinically actionable thresholds that can guide intervention without pathologizing normative connectivity.

Consider, for example, items such as “I cannot stand not having my smartphone,” which may conflate dependency with relational duty. The interpretive challenge is that such items fail to distinguish between individual compulsion and communal responsibility. We therefore propose that measurement invariance testing by *Ubuntu* adherence should reveal differential item functioning, and that culturally adapted instruments will reduce false positives without masking genuine dysfunction (Terblanché-Greeff and Nel, 2023; Olson et al., 2023).

## 9 African contextual determinants of smartphone use

Beyond philosophical orientation, material realities fundamentally alter the meaning of “intensive” smartphone use in African contexts. We identify four key contextual determinants that reframe high-frequency use as adaptation rather than pathology.

First, load-shedding and infrastructure instability mean that smartphones function as adaptive tools for safety, information, and coordination during power outages (Macleod et al., 2024). What might appear as constant phone checking in stable infrastructure contexts becomes a rational monitoring strategy when electricity supply is unreliable. Second, prepaid data constraints shape usage patterns in ways that Western frameworks fail to capture. Usage follows data availability, and families often employ collective strategies and compression practices to maximize limited resources (IGF 2017 Panel (WS188), 2017). This creates usage patterns that are episodic and collectively negotiated rather than individually compulsive.

Third, communal device-sharing and the preference for voice notes support intergenerational connectivity where literacy varies (Nguyen, 2020). A single device may serve multiple family members, making individual-level addiction assessments inappropriate and potentially misleading. Finally, economic survival functions require instrumental high-intensity use that is distinct from addiction. Banking, informal trading, and job access all depend on smartphone connectivity (Probst, 2015). However, Rammutla (2024) complicates this proposition by highlighting the increasing prevalence of online gambling among the unemployed and breadwinners, reminding us that economic precarity can intersect with genuinely problematic use patterns.

Taken together, these contextual realities reframe intensity as adaptation and relational coordination rather than necessarily indicating compulsion. This suggests that any assessment of problematic smartphone use in African contexts must account for these structural determinants before attributing observed behaviors to individual pathology.

## 10 Research agenda

The propositions and contextual analysis presented above point toward three interconnected lines of inquiry that could advance more culturally grounded understandings of smartphone use in African contexts.

### 10.1 Measurement development: *Ubuntu* smartphone use scale

The first priority is developing a measurement instrument that can distinguish relational connectivity, communal responsibility, collective identity, and contextual adaptation from genuinely problematic use (Terblanché-Greeff and Nel, 2023). This *Ubuntu* Smartphone Use Scale (USUS) must be developed through participatory processes that center African scholars and communities (Phiri et al., 2023), ensuring that the constructs measured reflect lived experiences rather than imposed frameworks. Validation should include multi-group invariance testing across urban and rural settings, age groups, and levels of *Ubuntu* adherence. Criterion validity must be established against meaningful outcomes such as bond quality versus interference in role performance. Comparative studies with existing instruments like the SAS and SAS-SV will be essential for demonstrating both the limitations of Western scales and the added value of culturally grounded measurement (Kwon et al., 2013; Mostert, 2025; Rotzinger et al., 2025).

### 10.2 Contextual investigation: mixed methods

Second, we need contextual investigations that employ mixed methods; combining surveys, narrative inquiry, and community-based participatory research (Khumalo and Nwoye, 2025; Mugumbate et al., 2023); to understand how *Ubuntu* values shape smartphone use practices. Key questions include: How does *Ubuntu* adherence shape responsiveness rhythms and expectations around availability? How do diaspora families sustain transnational relationality via WhatsApp, and what role does this play in maintaining *Ubuntu* values across distance (Bakuri and Amoabeng, 2023)? How do infrastructure challenges and data scarcity modulate the boundary between adaptive coping and genuine distress (Macleod et al., 2024; Probst, 2015)? And critically, do prevalence estimates of problematic smartphone use differ when using adapted versus Western scales (Soraci et al., 2024)? These questions require methodological approaches that can capture both the structural constraints and the cultural meanings that shape smartphone use patterns.

### 10.3 Intervention science: *Ubuntu*-aligned programs

Finally, intervention science must shift focus from individual self-regulation to family, community, and structural interventions (Brand et al., 2019). Rather than targeting the individual user’s behavior in isolation, *Ubuntu*-aligned programs might include family response-window protocols that negotiate collective expectations around availability, intergenerational digital literacy initiatives that build capacity across age groups, and pooled data strategies that address resource constraints (Gwagwa et al., 2022; Matahela, 2025). Community-level interventions such as peer groups and dialogs that address collective norms around smartphone use may prove more effective than individual counseling (Udah et al., 2025). Moreover, policy advocacy for affordable data and reliable infrastructure should be recognized as intervention work, since these structural factors fundamentally shape whether intensive use becomes distressing or remains adaptive. Evaluation of such programs should prioritize relational quality, community participation, obligation fulfillment, and financial stress reduction rather than focusing narrowly on screen-time reductions, which may be inappropriate or even harmful as outcome measures in contexts where connectivity serves essential relational and survival functions.

## 11 Discussion

This Perspective has argued that WEIRD-anchored frameworks for problematic smartphone use risk fundamentally misinterpreting African relational practices as addiction. By reframing smartphone use through *Ubuntu* philosophy, we have advanced three core propositions: that extended family WhatsApp communication reflects relationality rather than pathology, that FOMO operates as communal obligation rather than individual status anxiety, and that Western instruments systematically misclassify *Ubuntu*-aligned behaviors. These propositions point toward a research program centered on culturally valid measurement and intervention that can distinguish adaptive relational connectivity from genuine dysfunction.

To support clinical decision-making, Table 3 maps three interpretive zones for smartphone use under the *Ubuntu* framework. Zone A identifies behaviors that Western PSU instruments may flag as clinically concerning but which *Ubuntu* analysis reframes as normative relational connectivity—the core theoretical contribution of this Perspective. Zone B identifies behaviors that both frameworks recognize as warranting clinical concern, though they approach this recognition through distinct analytical lenses; *Ubuntu*-informed intervention in this zone would supplement individual treatment with community-level accountability and support. Zone C is arguably the most clinically significant addition of the *Ubuntu* framework: it identifies smartphone use patterns that may fall below Western diagnostic thresholds yet nonetheless disrupt relational obligations in ways that community members themselves recognize as harmful. This zone is likely to be systematically missed by individualist assessment instruments, underscoring the need for community-engaged assessment processes alongside formal psychometric tools. Taken together, this mapping operationalizes the central claim of this Perspective: *Ubuntu* does not propose that all intensive smartphone use in African contexts is adaptive, but rather that the criteria for distinguishing adaptive connectivity from pathological use must be grounded in relational rather than individual units of analysis.

**Table 3:** Three-zone conceptual map: smartphone use interpreted through Western PSU and *Ubuntu* frameworks (see main text, Section 8.3).

Zone	Example behaviors	Western PSU reading	*Ubuntu* reading	Clinical implication
Zone A Reframed as adaptive	High-frequency family WhatsApp monitoring; rapid responsiveness to elders; distress when temporarily disconnected from family groups; communal device sharing; voice-note chains sustaining intergenerational bonds	Possible dependency, inability to disengage, withdrawal-like distress; may meet SAS/SAS-SV threshold	Relational duty fulfillment; *Ubuntu*-aligned connectivity behavior; adaptive response to communal role expectations	Not pathological under *Ubuntu* criteria. Confirm relational motivation and absence of ego-dystonia before registering clinical concern
Zone B Shared clinical concern	Compulsive gambling or pornography use reported as distressing and contrary to personal values; use that community members identify as disrupting care responsibilities; use producing severe household financial harm	Compulsive behavior, functional impairment, significant negative consequences at the individual level	Ego-dystonic use disrupting relational obligations; community-recognized harm; individual and collective distress	Warrants intervention under both frameworks. *Ubuntu*-aligned intervention supplements individual support with communal accountability and family-level engagement
Zone C *Ubuntu*-specific concern	Individually moderate use (e.g., passive scrolling) that systematically withdraws the individual from family obligations and communal rituals; data or in-app expenditure compromising household survival and care of dependents	May not meet threshold for pathological classification under frequency or individual impairment criteria	Disruption of relational obligations; community-recognized failure of role performance; harm extending to the relational network rather than the individual alone	Likely missed by Western instruments. Requires community-engaged assessment and family-level inquiry. May warrant community-based rather than individual-level intervention

SAS, Smartphone Addiction Scale; SAS-SV, Smartphone Addiction Scale – Short Version; PSU, Problematic Smartphone Use.

The practical implications for psychologists working in African contexts are substantial. First, clinicians and researchers must contextualize intensity within relational obligations rather than treating high-frequency use as inherently problematic. Second, assessment instruments need adaptation to collectivist norms, recognizing that responsiveness to family and community may be a cultural strength rather than a symptom of dependency. Third, structural constraints; including load-shedding, data scarcity, and economic survival needs, must be considered before inferring individual dysregulation (Probst, 2015; Olson et al., 2023). Without these contextual considerations, practitioners risk pathologizing adaptive behaviors and imposing interventions that undermine rather than support wellbeing.

Several limitations warrant acknowledgment. As a conceptual article, this work requires empirical testing to validate the propositions advanced here (Billieux, 2012; De-Sola De-Sola Gutiérrez et al., 2016). The enormous diversity within and across African contexts cautions against over-generalization; *Ubuntu* philosophy itself takes different forms in different communities, and patterns of smartphone use will vary considerably by urban versus rural location, socioeconomic status, age, and other factors (Mokoena, 2023; Rotzinger et al., 2025). Our emphasis on WhatsApp reflects its current dominance in African digital communication but should not preclude attention to other platforms and emerging technologies (Nguyen, 2020). Moreover, while we have critiqued Western frameworks, integration with selected models; such as the Interaction of Person-Affect-Cognition-Execution (I-PACE) framework, remains valuable when adapted to local contexts (Brand et al., 2019). Finally, intersectionality deserves more systematic attention than we have provided here. Gender, age, disability, and sexuality shape both smartphone access and the meanings of connectivity in ways that our *Ubuntu*-centered analysis has not fully explored (Cho et al., 2013; Crenshaw, 1989). Future work must examine how these dimensions intersect with collectivist values to produce diverse experiences of digital life.

## 12 Conclusion

This Perspective has demonstrated that the uncritical application of Western-derived problematic smartphone use frameworks to African collectivist contexts constitutes a measurement validity crisis with significant theoretical, clinical, and ethical implications. By centering *Ubuntu* philosophy as an alternative interpretive lens, we have shown that behaviors currently pathologized by individualistic assessment instruments represent normative expressions of relationality in settings where personhood is defined through the principle “I am because we are.”

### 12.1 Key takeaways

This analysis yields ten critical insights for researchers, clinicians, and policymakers:

**Cultural validity is not optional**: Assessment instruments developed in WEIRD contexts cannot be assumed valid in collectivist settings. Measurement invariance testing must precede any cross-cultural application of PSU scales.

**Frequency does not equal pathology**: High-intensity smartphone use does not automatically indicate addiction in contexts where connectivity serves essential relational and survival functions.

**Context determines meaning**: Identical behaviors carry entirely different psychological meanings depending on whether they occur within individualistic or collectivist meaning systems.

***Ubuntu* reframes FOMO**: Fear of missing out in collectivist contexts centers on failing communal obligations rather than individual status anxiety.

**Structural factors shape use patterns**: Infrastructure instability, economic constraints, and survival needs create usage patterns that appear excessive under Western criteria but are adaptive responses to material realities.

**Family systems matter**: In extended family contexts, the individual is not the appropriate unit of analysis. Family-level and community-level approaches are required.

**Measurement colonialism persists**: The imposition of WEIRD-derived constructs without cultural validation represents a continuation of colonial power relations in knowledge production.

**Genuine dysfunction exists**: Arguing for cultural validity does not mean all intensive use is healthy. Culturally grounded instruments are needed precisely to distinguish adaptive connectivity from genuine impairment.

**Intervention must be culturally aligned**: Programs targeting individual self-regulation may undermine relational competence. *Ubuntu*-aligned interventions should address family protocols, community norms, and structural constraints.

**African epistemologies must lead**: Valid knowledge about smartphone use in African contexts can only be generated by centering African philosophical frameworks and African scholars’ expertise.

### 12.2 Implications for organizations and practice

Organizations working in African contexts; including universities, employers, healthcare systems, and technology companies, can apply these insights through several concrete actions:

Universities and student services should reconsider policies that treat high smartphone use as automatically problematic. Rather than implementing screen time restrictions based on Western norms, institutions should develop contextually appropriate guidelines that distinguish between relational connectivity supporting students’ family obligations and genuinely dysfunctional use interfering with academic performance.

Healthcare organizations and mental health services should audit their assessment tools for cultural bias. Clinics using the SAS, SAS-SV, or similar instruments with African clients should acknowledge that these tools lack demonstrated validity in collectivist contexts and may generate false positives. Practitioners should supplement or replace these instruments with culturally grounded assessment approaches.

Employers developing workplace digital wellbeing policies should recognize that employees’ smartphone use may serve essential family obligations that cannot be simply deferred to non-work hours. More appropriate policies would create designated times and spaces for managing family obligations while maintaining productivity expectations.

Technology companies should design features that account for collectivist use patterns. Screen time tracking calibrated to Western norms may inappropriately stigmatize users managing extended family obligations. Features supporting collective data management and family group communication would better serve African users’ needs.

### 12.3 Actionable recommendations

To address the problems identified in this analysis, we propose ten actionable recommendations:

**Develop the *Ubuntu* smartphone use scale (USUS)**: Prioritize participatory development of a culturally grounded assessment instrument that distinguishes relational connectivity from individual compulsion.

**Conduct prevalence comparison studies**: Directly compare PSU prevalence estimates using Western instruments versus *Ubuntu*-informed scales in matched African samples.

**Train practitioners in cultural assessment**: Develop training modules for psychologists, counselors, and healthcare providers on recognizing *Ubuntu*-aligned communication patterns.

**Establish contextual assessment protocols**: Create clinical decision-making frameworks that systematically assess structural factors before attributing intensive use to individual pathology.

**Design family-level interventions**: Pilot and evaluate intervention programs that target family communication protocols and negotiate collective expectations around availability.

**Build community-based research partnerships**: Establish collaborations between universities and communities to co-create research questions, methods, and interventions.

**Advocate for infrastructure and data access**: Recognize that policy advocacy for reliable electricity and affordable data is mental health intervention work.

**Reform university ethics review processes**: Update research ethics protocols to explicitly address epistemic justice concerns.

**Create cross-cultural validity standards**: Professional organizations should establish standards requiring measurement invariance testing before instruments can be marketed for cross-cultural use.

**Center African scholars in knowledge production**: Funding agencies and journals should prioritize research led by African scholars working from African philosophical frameworks.

### 12.4 Strengths and limitations

This study’s primary strength lies in its theoretical originality and conceptual coherence. By systematically applying *Ubuntu* philosophy to problematic smartphone use, it offers a comprehensive alternative to Western frameworks. The integration of *Ubuntu* with established psychological theories provides bridges to mainstream science while preserving the distinctiveness of African relational ontology.

However, several limitations must be acknowledged. As a conceptual analysis, the study does not provide empirical evidence demonstrating that *Ubuntu*-aligned behaviors are non-pathological; these remain propositions requiring empirical validation. The diversity within and across African societies means *Ubuntu* philosophy is not uniformly endorsed or practiced. The study focuses primarily on WhatsApp due to its dominance in African digital communication, but this may limit applicability to other platforms. Gender, disability, and sexuality receive insufficient attention despite their importance in shaping both smartphone access and the meanings of connectivity.

### 12.5 Perspectives for future work

Future research should pursue several interconnected lines of inquiry. First, empirical validation of the propositions advanced here through mixed-methods studies combining quantitative measurement with ethnographic investigation. Second, systematic examination of within-Africa variation to identify how factors such as urbanization, education, and exposure to Western influences shape smartphone use. Third, investigation of how digital communication platforms can support or undermine *Ubuntu* values in diaspora contexts. Fourth, development and testing of *Ubuntu*-aligned interventions at family, community, and policy levels. Fifth, comparative research with other non-Western collectivist contexts to identify common patterns versus context-specific factors. Finally, critical examination of how global technology companies’ business models and design practices either support or undermine collectivist social ecologies.

Advancing epistemic justice in psychology requires centering African knowledge systems and co-creating models that honor communal identity and responsibility alongside individual wellbeing. Reinterpreting problematic smartphone use through *Ubuntu* philosophy represents one step toward culturally valid science that can improve both clinical practice and public health interventions. The challenge ahead is not simply to adapt Western psychology for African contexts, but to fundamentally reimagine what constitutes problematic use when relationality, rather than autonomy, is the organizing principle of social life.

## Data Availability

The original contributions presented in the study are included in the article/supplementary material, further inquiries can be directed to the corresponding author.
